# “Saving lives”: Adapting and adopting Human Papilloma Virus (HPV) vaccination in Austria

**DOI:** 10.1016/j.socscimed.2016.02.006

**Published:** 2016-02-06

**Authors:** Katharina T. Paul

**Affiliations:** University of Vienna, Department of Political Science, Universitätsstrasse 7, 1010 Vienna, Austria

**Keywords:** Austria, HPV, Vaccination, Policy, Gender, Problematization

## Abstract

Vaccination against the sexually transmitted Human Papilloma Virus (HPV), a necessary agent for the development of cervical cancer, has triggered much debate. In Austria, HPV policy turned from “lagging behind” in 2008 into “Europe's frontrunner” by 2013. Drawing on qualitative research, the article shows how the vaccine was transformed and made “good enough” over the course of five years. By means of tinkering and shifting storylines, policy officials and experts disassociated the vaccine from gender, vaccine manufacturers, and youth sexuality. Ultimately, the HPV vaccine functioned to strengthen the national immunization program. To this end, preventing an effective problematization of the extant screening program was essential.

## 1. Introduction

In August 2013, the Austrian Minister of Health, Alois Stöger, announced the introduction of a vaccine against the sexually transmitted Human Papilloma Virus (HPV), a necessary agent for the development of cervical cancer. It would be administered to all 9-year old children, making Austria a frontrunner in HPV vaccination policy, compared to its European counterparts where only girls were being vaccinated. Yet those countries had adopted the vaccine five years earlier, as EU level expert committees had recommended (ECDC, 2008).

This paper follows a lengthy, and not always public, debate on HPV vaccination in Austria. We trace how HPV policy turned from “lagging behind” in 2008 into “Europe's frontrunner” in 2013. In doing so, we find interesting parallels to historical efforts to turn the Pap test into the “right tool for the job” of cervical cancer screening (Casper and Clarke, 1998). Having observed the initial rejection of the vaccine in 2008, we ask: How did the HPV vaccine become *good enough* in Austria?

In exploring this process, we draw on in-depth interviews with policy officials, clinical experts, researchers, civic actors, and members of the pharmaceutical industry (n = 13), documents (n = 100+), and observations at public events (n = 8) pertaining to vaccination. We present our analysis in three inductively derived stages of translation (Callon, 1986): *problematization*, *enrollment*, and *embedding*. Below, we first review the existing scholarship and then lay out our analytical framework.

## 2. Cervical cancer prevention: issues and debates

Cervical cancer is the second most common malignancy and cause of cancer-related death in women worldwide (Boulet et al., 2008). In Europe, despite widespread and organized gynecological screening programs employing the Pap smear test, named after its inventor Georgios Nicholas Papanicolaou, cervical cancer remains the 10th most common cause of cancer-related mortality among women (Boulet et al., 2008). In the late 1990s, medical research made for global headlines when it concluded that infection with specific strains of HPV could cause cervical cancer. Women in good health largely fight off HPV as well as precursors to cervical cancer, lesions known as cervical intraepithelial neoplasia (CIN) 1, 2, and 3, but chronic HPV infection is responsible for over 90% of cervical cancer incidence. In 2006 and 2007, respectively, two vaccines (*Gardasil* and *Cervarix*) were approved by the United States Food and Drug Administration (FDA) and the European Medicines Agency (EMA). These vaccines were intended to immunize girls and women against several strains of HPV, targeting specifically those that cause cervical cancer (strains 16 and 18, 31 and 33), and, in the case of *Gardasil*, additionally those that cause genital warts (6 and 11).

The medical research community heralded this breakthrough as the “first vaccine against cancer” (Gericke, 2008), recalling earlier hopes to “eliminate the need for screening” (Richart, 1995: 1926). Conversely, critical observers commented on the vaccine as an uncertain and costly tool that might either sexualize children at an early age, or medicalize women unnecessarily who might benefit more from improved Pap-based screening programs.

To be sure, state-sponsored vaccination programs have frequently triggered political conflicts (Colgrove, 2006), be it the pertussis vaccine (against whooping cough) in the 1970s (Blume, 2006), measles, mumps, rubella (MMR), the vaccine against H1N1 (‘swine flu’), or Hepatitis B. These conflicts have typically been articulated around notions of risk, excessive government interference versus personal autonomy and parental control (Reiter et al., 2009; de Visser and McDonnell, 2008), violations of children's bodies, public versus individual health, and of definitions of what it means to be healthy (Hardon and Blume, 2005). Similarly, vaccination policies embody tensions between eliminating a disease, on the one hand, to achieve ‘herd immunity’, and protecting individual choice, on the other, particularly in what Reich (2014) refers to as neoliberal cultural frames. The tension between these imperatives is particularly prone to political mobilization when a condition is seen as the result of a risky lifestyle (cf. Lupton, 1995: 50ff) and individual (ir)responsibility. HPV vaccination is a case in point.

In recognition of another central political tension, feminist scholars and health advocates have viewed the HPV vaccine in relation to *biopolitics*, through which women's lives are brought under medical surveillance (Carpenter and Casper, 2009; Mishra and Graham, 2012; McKie, 2008) in a moral framework of self-responsibility and social obligation (Howson, 1999: 401;Armstrong 2007). Indeed, immunizing young children and adults against HPV became highly controversial across countries (Wailoo et al., 2010) and representative of what Mamo and Epstein (2013) conceptualize as a “pharmaceuticalization of sexual risk”, much like the associations of gay sex with HIV/AIDS in the 1980s, which linked sexuality and disease in novel ways.

These new associations and tensions came to question existing screening and prevention policies. As we shall see, the competing public health logics of screening and primary prevention and the related tools – Pap and vaccine – as well as their designated administrators came to be central tropes in Austrian HPV policy.

## 3. Analytical framework and methodology

The review above reveals a need for empirical observations of HPV policy *in the making*. The framework developed by Casper and Clarke (1998) in their study of the emergence of the Pap smear is helpful for this purpose. They show that, in the 1960s, the Pap smear did not just emerge as the “right tool for the job” but had to become “good enough” and *embedded* in particular social and material work arrangements (Casper and Clarke, 1998). It had to satisfy several “social worlds” involved in cervical cancer screening, including medical professionals, technicians, insurers, and women's groups. Our framework similarly draws on Timmermans and Berg's proposal that “technologies are embedded in relations of other tools, practices, groups, professionals, and patients and it is through their location in these heterogeneous networks that […] action is possible in health care” (2003: 104). Methodologically, this means considering “technology in practice” (Timmermans and Berg, 2003) *and* the social worlds (cf. Clarke and Star, 2008) that technologies enable, contain, reproduce, and embody.

Ontologically, we draw on Science and Technology Studies (STS) and feminist approaches to technology (Wajcman, 2008); we view technologies as contingent on and inseparable from their social context (Jasanoff, 2004). We opt for an asymmetrical approach in our *situational analysis* (Casper and Clarke, 1998; cf. Hogarth et al., 2012), in which we choose to focus on the claims and practices of a range of social agents. This stance enables us to investigate notions of ‘goodness’ and ‘rightness’ (of time, space, agency, and tools) as artifacts in their claims. By extending the concept of *situation* (Clarke and Fujimara, 1997) to include *policy* infrastructures – in our case, the national immunization program (NIP) – we argue that, when a new technology emerges in a particular context, its introduction involves tinkering with the very design of the technology *and* the *infrastructure* in which this technology emerges to make it “good enough”. Borrowing Clarke's and Fujimara's (1997) terms, we argue that tools, jobs, and rightness are co-constructed in health policy infrastructures, too, and *embedded* therein.

We inductively distilled three – in part overlapping - phases of HPV policy in Austria from our material and conceptualize them as moments of *translation* (Callon, 1986). At the stage of *problematization*, an object moves (or is being moved) towards becoming indispensable and key agents attempt to define the nature of the problem and the roles of other actors to fit the proposed solution. In the process of *enrollment*, roles are coordinated, which helps establish a stable network of alliances. Enrollment is only successful, however, if actors impose their will on others. In our case, the translation entailed *problematizing* historically contingent screening infrastructures and their gatekeepers, *enrolling* social worlds pertinent to the NIP, and what we term the (ongoing) *embedding* of the vaccine, drawing on our analytical argument above.

STS provides little methodological guidance to account for successful enrollment or “coproduction” of technologies and social orders (Jasanoff, 2004). We therefore draw on discourse analysis and identify “storylines” as key tools for enrollment and embedding: “A storyline guides and shapes a policy process over a period of time […] it allows actors to develop the story, to change it according to new insights or to fill in the blanks over time” (Hajer, 2009). Storylines typically remain reasonably open and do not as such imply consensus, but function to conceal past dissent. By identifying storylines, we highlight both conflict and its concealment in vaccine *adaptation* and *adoption*.

Our methodological choices and the resultant account must be understood in context: Whereas coverage of North American HPV controversies has often relied on pharmaceutical campaigns and health activism, Austria features a federalist social health insurance system as the most important source of financing. Vaccination in the NIP is tax-funded and direct advertising of pharmaceutical products is forbidden. Policymaking can be described as corporatist and health activism is scarce. In our analysis, we draw, first, on qualitative in-depth interviews (n = 13) with officials at the Ministry of Health (MoH), members of the pharmaceutical industry, researchers, clinical experts, feminist health activists, and professional associations. We employed the snowball method to obtain a sample of what proved to be a fairly closely-knit policy community. Upon oral consent of respondents, most interviews were recorded and subsequently transcribed verbatim. Interviews lasted 75 min on average. We used individual interview guides in these conversations and analyzed their content for repetitive themes, references to key events and positioning of other agents. Second, we consulted pertinent written documentation (n = 100+), meeting reports, and press releases issued around key moments, as identified by interview partners. Third, throughout the study, we attended public events (n = 8) pertinent to the subject matter in order to get a sense of the elite discourse on health policy and vaccination. These events proved helpful in identifying potential respondents and allowed us to observe the changing nature of the debate and the ways in which HPV policy was referred to (for instance, as a failure or a success). We also subscribed to newsletters of government agencies and civic actors, and followed national and international science journalism. Finally, we subscribed to a web alert (Google alert) listing the terms “vaccination” or “HPV” in both English and German, and subscribed to a digital service (OTS) delivering national press releases containing items with the term “health” or “health policy”.

The analysis of secondary literature, interview transcripts, and observational notes took the shape of situational and positional maps (Clarke and Star, 2008). These maps included a range of diachronic subjects, ranging from cancer research and the cells obtained from Henrietta Lacks without consent (Löwy, 2011), to the HPV vaccine, social-scientific as well as epidemiological concepts, and social agents. During the writing up of our analysis, it became increasingly clear that time was a key aspect, specifically the ways in which respondents discussed their agency in terms of anticipation, surprise, innovation, and rightfulness of (non-)action. Therefore, we chose to present our empirical narrative in a chronological fashion.

## 4. Translating cervical cancer prevention in Austria

### 4.1. Problematizing screening practices 2006–2008

The HPV vaccine *Gardasil* was licensed comparatively early in Austria, notably for girls and boys, in October 2006, against a rather particular cervical cancer screening infrastructure that warrants explanation. In Austria, a nation-wide cervical cancer screening strategy was first set up in 1970. Women above the age of 20, or at the latest two years after commencing sexual activity, are offered annual screenings, though remarkable variation exists even within Austria (e.g. Krebshilfe 2014; Hauptverband, 2014). Despite the early introduction of the program, it has remained flawed due to its loose and opportunistic character: participation depends on individual initiative rather than a national recall system. Estimates suggest an annual screening participation of around 30% (Hauptverband, 2007). Its effectiveness is additionally hampered by variation in the tools used and insufficient communication between gynecologists, cytologists, and technicians (Rásky, 2006). This enhances the probability for mishaps, known as “bad smears”. Intersubjective reading variation and persistent shortcomings in sensitivity and specificity further hamper diagnostic validity (Boulet et al., 2008: 11, 13–15). In Austria, much of quality assurance is left to professional associations, reflecting a high degree of professional autonomy. Moreover, only specialists may perform the test, quite in contrast to other EU countries, where GPs or specialized nurses may obtain the smear sample. Despite its flaws, the professional stakes invested in the Pap test were thus high.

At the same time, the advent of the HPV vaccine was welcomed with a sense of innovation in gynecology. Prominent gynecologists reportedly anticipated that the vaccine would “revolutionize gynecology […] [and we would] no longer have to worry about the whole quality assurance question around the Pap test” (Interview 10). *Gardasil* saw one of the speediest licensing trajectories ever in Austria: clinical trials by the manufacturer were ended early, because the vaccine's prophylactic effects had been exceptionally clear (Interview 10). It is worth noting that, at the Medical University of Vienna, a group of gynecologists and gynecological oncologists, as well as dermatologists had been intensively studying HPV and vaccine effectiveness since 2001, publishing very successfully on the subject matter (e.g. Joura et al., 2007, 2012).

The vaccine therefore presented a double-edged sword for those social worlds invested in reducing, if not preventing, cervical cancer in Austria: the HPV vaccine appealed to gynecologists and the MoH, particularly the officials in charge of vaccination policy at the time. Yet it also brought about a potential to problematize the national screening program, particularly its opportunistic character, the lack of quality assurance, and the fragmented nature of its organization. Fragmentation, in this context, relates to Austrian federalism. Despite Austria’s small size, its federal states share competencies with the federal government in a range of policy areas such as public health and education. In fact, the NIP is exemplary of this complexity: Since 1998, the NIP has been jointly funded by federal states (1/6th), federal government (4/6th), and the social insurers' federation (1/6th). As a cornerstone of the Austrian public health system, the NIP is based on equity regardless of income, nationality, gender, or other categories. Importantly, vaccination is not mandatory.

The following presents an account of how these issues were mitigated in the process of adopting– and, as we shall see, *adapting* – the HPV vaccine for the NIP. Following the approval of the vaccine in 2006, the Minister of Health asked the Supreme Health Council (*Oberste Gesundheitsrat*, OSR) to assess the possibility of vaccine introduction. The OSR is a fairly intransparent and, as we found in our research, inaccessible committee, that, contrary to some of its European counterparts such as the German STIKO and the Dutch *Gezondheidsraad*, does not publish minutes and extensive reports. The OSR operates independently of the MoH, but allows the assignment of in-house Ministry experts. Moreover, the Ministry features its own vaccination expert committee, which operates “in close collaboration” with the OSR vaccination committee. This setup indicates that feasibility and goodness of fit in the NIP are discussed at a very early stage. Its recommendations are published annually.

In September 2007, the OSR issued a recommendation in favor of including HPV vaccination for girls and boys in the NIP. It was then up to these experts to convince the Minister at the time, Andrea Kdolsky, that the vaccine was “good enough”, if not indispensable, for the NIP. Given the comparatively high costs of the HPV vaccine, and its novel feature as a vaccine against a carcinogenic virus affecting women primarily – this was the state of knowledge at the time - the MoH faced a multiplicity of new diagnostic and therapeutic options, as well as potential conflicts. While the key officials formulating policy proposals were in favor of expanding the NIP with the HPV vaccine (Interview 1, 2), Minister Kdolsky, a Conservative and a medical doctor herself, expressed skepticism regarding the usefulness of the vaccine, as it might endanger the national screening program (BMG, 2007). Cervical cancer incident rates, the Minister was concerned, might in effect rise, rather than fall, with the introduction of the vaccine.

While the ECDC (2008; cf. Raffle, 2007) had similarly raised concerns about possible lower attendance to screening programs as a result of vaccine introduction, Kdolsky's stance presents a somewhat idiosyncratic interpretation of these concerns. She contested the message communicated by medical professionals and patient associations, such as the *Krebshilfe*, that “this vaccine could prevent cancer”, as, in fact, it was the *virus* that caused cancer, and the vaccine would protect against the virus, rather than *cancer*. But more importantly, she asserted: “Efficacy and safety form top priorities, and no evidence-based data and long-term studies are available at this point. We do not know […] the effects of administering retro-viral, that is, gene-technology based inactive cells” (BMG, 2007). Against the background of a history of resistance against genetic modifications in Austria (Felt, 2013; Prainsack et al., 2010), Kdolsky's reference to genetic technology carried some weight in this context and was likely to provoke more criticism than support for the vaccine.

The inexplicable death of a young girl in October 2007 in Upper Austria only a few weeks after her first HPV vaccination dose added further fuel to the emerging debate (cf. Stöckl, 2010, Interview 1, 7). There was a risk that historical skepticism towards vaccination (particularly against MMR) in sub-communities would gain new momentum. While empirical data regarding this skepticism is limited, most respondents refer to it as a given. Notably, in Austria, anti-vaccination sentiment is not primarily religiously driven, but it includes views of vaccinations as interventions in the natural course of childhood, fears of “unnatural” adjuvant substances, such as aluminum (Interview 1), and, as a recent survey suggests, widespread beliefs that vaccination may cause allergies (OTS, 2013).

Beyond the safety concerns discussed above, budgetary issues and the industry's willingness to lower prices formed primary concerns in the Minister's argumentation against an immediate adoption of the vaccine (BMG, 2007). This focus on costs and uncertainty, we propose, may also reflect her reservations as a Conservative to carry responsibility for a vaccine that touched upon the taboo subject of youth sexuality. As mentioned above, in Austria, girls are transitioned to specialist gynecological care for the Pap test relatively early, as a result of which sexuality is compartmentalized as a medical specialist matter at a comparatively early stage.

The Minister found herself in an emerging mediatized debate that could potentially harm two cornerstones of public health: cervical cancer screening *and* the NIP. She was to reject the OSR advice, based on her concerns regarding safety and effectiveness, but needed additional political support to remain credible and independent of other social worlds invested in Austrian HPV policy *in the making*. In an attempt to depoliticize the emerging debate by demonstrating objectivity, she commissioned an “independent economic evaluation” (Interview 11) of the vaccine from a newly founded research institute for Health Technology Assessment (HTA). While HTA is common practice in other EU countries' policymaking, assigning numerical *value* to, for instance, the number of lives saved per year by means of a public health intervention, was comparatively unusual in Austria at the time. Yet it appeared to fit well with a fairly managerial Minister and her political party, which tends to prize economic expertise. As a multidisciplinary assessment, but also a form of ‘gating’ practice (Black, 2013), HTA considers aspects such as safety, efficacy, effectiveness, necessity, economic efficiency, social impact, and equity. Commissioned by the Minister, the HTA report – the first of its kind in Austria - had to be completed within three months, by the end of 2007, and had to focus on a purely economic evaluation. The HTA report resulted in three options (Zechmeister et al., 2007: 17):

Option 1: Improvement of screening, that is, optimization of preventive measures.
Option 2: Vaccination under improved cost-effectiveness due to lower vaccination price.
Option 3: Vaccination under current condition, but with a high risk of uncertainty and high costs.

The Minister of Health ultimately chose option 1. The vaccine was made available in pharmacies for private purchase (around 600EUR for three doses) but not included in the publicly funded NIP. Yet as the report has been criticized by many for its simplifications (Interview 1, 2, 7, 8) and was referred to as a key moment by nearly all respondents, it is worth exploring it in some detail. First, in addition to its exclusively economic focus, the evaluation was driven by situated local assumptions and conditions, such as poorly available data on screening effectiveness and vaccine uptake. Second, the report only estimated cervical cancer prevention rates, excluding condyloma and cancers affecting men, such as anal and penile cancer, as the vaccine had not been studied in men yet. Neither did the report take into account the effect of the vaccine in preventing pre-cancerous lesions and costs associated with an increase in miscarriages following their removal in conizations (Interview 7).

Third, critics have questioned the comparatively high discounting rate used in the assessment (5%, rather than 3%) (Interview 7, 1). This methodological choice, along with a high price assumption, resulted in comparatively conservative projections of vaccine effectiveness (10, rather than 20 years) and cost-effectiveness. As governments purchase vaccines at a significantly lower rate than the official retail price, this was a particularly sensitive assumption. Finally, neither policy officials nor the commissioned HTA report at the time addressed sexual activity, which meant leaving out all risks of HPV to both male and female partners, regardless of their sexual orientation, much like vaccine developers had done (cf. Fisher and Ronald, 2010). Notwithstanding the potential flaws in the analysis, we propose that they reflect not so much ‘flawed science’, but *local* science, and were informed by an economically oriented ministerial order, limited specialized expertise, and a single consensus document, which, at the time, formed the basis for HTA practice in Austria (Walter and Zehetmayr, 2006; cf. Zechmeister et al 2007, fn 58).

Let us return now to the three suggested policy options. Politically speaking, option 3 was clearly unattractive and not recommended, and potentially appealed to the more general historical skepticism of vaccination in Austria discussed above. Option 2 was costly as it would involve negotiating with the bearers of the costs of the NIP and conceding to other stakeholders' demands. Option 1, arguably vague, non-specific, and not immediately costly (neither financially, nor politically) seemed most attractive and would at once protect the Pap infrastructure and its gatekeepers. In sum, the report therefore helped stabilize a storyline that still featured *women* in need of protection, yet not only against cervical cancer, but against an uncertain new vaccine.

Feminist critics of the vaccine could relate to option 1 and its underlying storyline, too, both in their resentment of the medicalization of women's bodies and their concerns regarding vaccine manufacturers' bias (Interview 6). Their concern regarding the apparent profit-seeking behavior of vaccine manufacturers and the bias in clinical research sponsored by vaccine manufacturers also resonated in the tone of media reports at the time (Stöckl, 2010). At the same time, their suggestion that campaigns exaggerated cervical cancer rates to promote vaccine sales, again excluded men's health as a public health issue, much as the HTA report had done. This exclusive focus on women around a shared storyline becomes visible, for instance, in a lead article written by the head of a feminist health center (*Frauengesundheitszentrum*), published in the HTA research group's newsletter (Groth, 2007). The article calls into question the long-term effectiveness of the vaccine, its use for women previously exposed to HPV, and demands the set-up of a standardized nation-wide vaccination register next to an improvement of the Pap smear in Austria, instead of adopting the vaccine.

Against this association of feminist, economic, and safety-related claims, protecting the *status quo* was ultimately the safest option, and the Austrian debate around the HPV vaccine was virtually closed at the level of public health policy. At the same time, the short-lived media controversy around HPV brought about a clearer alliance of proponents of the vaccine: clinical experts and researchers at Vienna Medical University, individual policy officials at the MoH, and experts at the OSR. While they had to concede to HPV vaccine critics, and the Minister's decision, at this stage, it was important to avoid further problematization of either screening or the NIP. Appeasing earlier criticism, the Austrian social insurers' federation (*Hauptverband der Sozialversicherungsträge*r) thus launched an evaluation project of the Pap smear (“QUOPAP—Qualitätsoffensive Pap-Abstrich”; cf. Rásky, 2006; Rasky et al., 2013). In addition, and quite in line with the political segmentation and high degree of professional autonomy in Austria, the Viennese Sickness Fund (WGKK) launched a separate quality improvement project in collaboration with the Viennese medical association. Finally, the Austrian Cytology Association launched a self-monitoring initiative, too.

To conclude, the detailed analysis of the HTA report and the political context of its production show that the chronic *instabilities* of the Pap smear (Singleton, 1998) were indeed re-exposed and *problematized* with the advent of the HPV vaccine. Yet the early HPV debate in Austria was rapidly closed by bringing forward arguments of cost-effectiveness to thwart a broader debate on the quality of the existing screening program or the weaknesses of the NIP in this politically, institutionally and professionally fragmented context.

### 4.2. Enrollment: remaking the vaccine and its context 2008–2013

Following the Minister's decision not to include the HPV vaccine in the NIP, the debate seemed virtually closed at the level of federal health policymaking in Austria. While vaccine developers continued to tinker with, trace and study the HPV vaccine and its features, it was making its way into Austria's public health system, using a different route. First, growing evidence concerning the carcinogenic nature of various strands of HPV led to studies suggesting promising effectiveness for the prevention of anal cancer, rates of which had been rising in both women and men who have sex with men. A multinational trial of Gardasil was conducted in men, and in the US, the HPV debate began to endorse the notion of vaccinating boys, too, even if campaigns were by no means as prominent as they were for girls and women (Epstein, 2010: 72ff). Furthermore, head and neck squamous cell carcinoma (HNSCC), a disease largely attributed to environmental exposures, were found to be preventable by way of vaccinating against some strands of HPV.

Second, in Austria, regional experiments with subsidizing the vaccine were initiated in 2007 and 2008 and continued in Lower Austria, Burgenland, and Vorarlberg, enrolling local support. Third, the vaccine's supporters – key MoH officials, clinical researchers - regrouped around a new storyline as a means to enroll actors: “saving lives” and “fighting cancer”, two prominent notions in contemporary health policy. Encompassing these notions, the discourse of prevention gained increasing prominence as “the most important pillar for a sustainable health policy” (BMG, 2013). This appeal to prevention and a collective future was an effective storyline to invoke coherence and mutual interests. At the same time, the persistent feminist critique was positioned as “against the HPV vaccine” and even “vaccination in general” (Interview 1, 7). In this way, feminist voices were quelled, and their persistent critique of the HPV vaccine was presented as irrational, as questioning the NIP *in general*, and even as “against their own interest” (Interview 1). This positioning effect continued over the years, and led to mediatized conflicts between clinical researchers calling for gender-neutral HPV vaccination and public health researchers skeptical of long-term effectiveness in late 2012. Notably, the latter shared a key concern with feminists, namely the call for introducing harmonized vaccination registers in Austria prior to introducing any new vaccines. Thus critics of the vaccine were at the same time critics of the infrastructure in which it was to be embedded, indicating the inseparability of technology and context.

In turn, key policy officials in charge of vaccination at the MoH invested in solidifying this infrastructure to counter further *problematization* of the NIP. Now headed by a new Minister, Alois Stöger, considered generally in favor of HPV vaccination (Interview 1), the MoH continued to strengthen the NIP by adding two – notably less controversial – vaccines in 2012, against pneumococcal and meningococcal infections. Another key move was a more visible construction of the need for the HPV vaccine: Statistical surveillance of HPV infections in Austria continued and began to explicitly – and equally – refer to women and men, and all cancers caused by HPV (e.g. Statistik Austria 2009, cited in BMG, 2013: 2). This meant that the vaccine was being disentangled from women's bodies alone and helped construct a *public*, rather than gendered need for the vaccine. Again, from a discursive point of view, the construction of a collective problem and a solution at once contributed to a persuasive storyline to enroll a broader range of social worlds.

In addition, officials at the MoH formed new collaborative networks with pharmaceutical manufacturers in the shared aim to promote vaccination uptake. Specifically, members of the industry coalesced in the new Austrian Association of Vaccine Manufacturers (ÖVIH), the self-declared aims of which include an “evidence-based political discourse on vaccination” (ÖVIH, 2015). Interestingly, its slogan appeals to a shared responsibility, a recurrent theme in vaccination policy, not least in Austria: “Vaccination means carrying responsibility for the individual and society at large” (ÖVIH/BMG/AGES, 2011). In related networks, a first survey was conducted on parental decisions in Austria regarding vaccination and their concerns, suggesting poor supply of information regarding vaccines and considerable distrust in them (OTS, 2013). This type of data collection further contributed to the construction of the need for a stronger NIP.

At the level of the EU, data continued to be gathered on cervical cancer and HPV vaccination (ECDC, 2012), continuously listing Austria as an outlier case in HPV policy. Respondents (Interview 1, 7, 10) anecdotally refer to a sense of embarrassment vis-à-vis EU counterparts, as well as countries in the Global South that had successfully launched mass-vaccination. Meanwhile, Vienna-based researchers continued to investigate vaccine efficacy, publishing widely and updating policy officials. These practices not only contributed to the construction of a *need* for the vaccine, but also helped form the conditions of possibility for its adoption.

Next to campaigns, support for the vaccine was mobilized at particular events. In October 2012, the MoH gathered Austrian experts in an exclusive seminar in Vienna, where clinical experts from the UK presented new data on HPV. This evidence, along with the simultaneous publication of an important review article regarding the vaccine's effectiveness (Schiller et al., 2012) meant that the data gathered was now not only enough, but also *good enough*, as it pointed to the usefulness of vaccinating both boys *and* girls, thereby transforming it into a less gendered vaccine. This tinkering helped to depoliticize the vaccine and made it an easier fit for the NIP, which historically rests on the principle of equity, perhaps now even more so under a social democratic Minister.

Furthermore, evidence from Swiss trials revealed that administering two, rather than three doses, offered sufficient protection, making the vaccine less of a burden organizationally. Two doses could be completed more easily within a school year; therefore, this presented yet another step in reworking both vaccine *and* infrastructure for its institutional embedding. At the same time, the price of the vaccine had decreased significantly, while its prophylactic spectrum had broadened and it had already been licensed for administration to children as young as 9 years old: between 2007 and 2013, the HPV vaccine had (been) transformed into a vaccine that was very different from the product introduced in 2006.

In August 2013, in the midst of what is typically a time of the year without much political news, the Minister of Health, Alois Stöger, announced: “We have expanded the free children's vaccination program step by step. I am certain that, with the inclusion of the vaccine against HPV, we will be offering an essential contribution to the health of our children. We will save lives.” (BMG, 2013). Adding to the effectiveness of this storyline, an expert team presented the decision as univocal and beneficial to all: the Minister of Health, Alois Stöger, Pamela Rendi-Wagner, director of public health at the MoH and immunologist by training, and the President of the patient association *Krebshilfe*, Paul Sevelda, who also practices gynecology. The minister announced that the vaccine – *Gardasil* – would be offered to children as young as 9 years old. This enrollment effected that the vaccine could be disassociated from the Pap test, and from gynecologists and clinical researchers receiving grants from vaccine manufacturers, who had been suspected to be insufficiently objective. It could be placed in the hands and offices of a professional group typically considered trustworthy: pediatricians. In this way, the final step of *adaptation* was taken: the vaccine had become desexualized, as pediatric settings and primary schools are typically not associated with sexuality.

### 4.3. Embedding the vaccine

The embedding of the vaccine in the NIP, particularly given its transformation into a gender-neutral prophylactic vaccine, was not quite as smooth and immediate as the press conference had insinuated. A first potential problem that was elegantly glossed over is the fact that school doctors are not obliged to – nor insured for – vaccinating children in federal public schools (*Bundesschulen*). This legal aspect is not publicly debated, but nonetheless a political problem, a respondent recounts (Interview 13), as school headmasters tend to be informally associated with particular political views on vaccination, and uptake hence depends on them, too, to a certain extent. A second issue quickly taken up by critics concerned the need to vaccinate within a school year to ensure completion rates, yet this was speedily fixed by postponing the original starting date and offering vaccination at public vaccination offices, too, until a child's 12th birthday. This means that schools, as well as pediatricians, mostly found in private practices, and GPs offer the vaccine. Crucially, this also means that direct parental consultation can be circumvented in school-based vaccination programs. Parents or caretakers of children under the age of 14 are provided with a brief health status questionnaire and a consent form. Yet any questions or concerns children may have regarding HPV and sexual transmission are compartmentalized to a professional pediatric setting, much like the Pap smear is in gynecological practices. Leaflets used for the required parental information explain a wide spectrum of possible effects of chronic HPV infections and call for preventing “all tumors associated with HPV infection” before referring to cervical cancer at all, ensuring gender-neutrality. Furthermore, the leaflet explains that “the disease-related viruses are transmitted by direct mucous membrane contact, such as by sexual contact or from the mother to the child during birth” (BMG, 2015: 1), therefore backgrounding sexuality as much as possible.

This enrollment and embedding of the vaccine to “save lives”, rather than only “women's lives”, also came with costs for some social worlds involved in the translation trajectory: Gynecologists were largely disassociated from HPV policy, even though their long-term involvement in studying the vaccine entailed a high level of prestige and sense of ownership (Interview 7). Moreover, demands by critics of earlier attempts to include the vaccine in the NIP, namely the introduction of national vaccination registers, have not been met: The MoH continues to rely on estimates calculated by the federal states in diverse ways, a practice rather unusual for Europe and not quite in line with the recommendations of the ECDC and the WHO. By positioning critics – be it feminists, economists, individual politicians, or public health scholars – as (irrational) *vaccination oppositionists*, attention was diverted not only from a potential debate on youth and sexual health, but, at this stage, steered away from the *weaknesses* of the NIP towards the *strengths* of the NIP.

We were able to observe this appeal to a shared storyline at a series of events. Regional “Vaccination Days” have historically served to educate and update doctors, pharmacists, and students regarding vaccination, and these were typically co-sponsored by the pharmaceutical industry. In 2014, however, the first “Viennese Independent Vaccination Day” took place. At the event we attended in January 2015, presenters and moderators alike emphasized their independence from the pharmaceutical industry. HPV vaccination was presented as a great success, and particularly the “gender neutral” nature of the HPV vaccination program was presented as an exceptional and progressive feature of the NIP. The fact that, regarding inclusion in the NIP, Austria was not quite an early adopter was successfully concealed, and HPV vaccination was celebrated as a particular success.

We observed another instance of concealment of dissent at an event hosted by the National Bioethics Commission in April 2014. The event featured a skillfully staged sequence of talks by officials, foreign bioethicists (suggesting enhanced independence), and even parents of a deceased son who had passed away from secondary complications of measles and who had attended an anthroposophist school, which is typically associated with opposition to vaccination in Austria. Similarly, at a conference organized by the research platform on Ethical and Legal Aspects of Medicine and, interestingly, hosted by the Viennese Medical Association in September 2014, HPV vaccination was articulated as an indispensable element of the NIP, and an expert composition of historical, legal, and medical presentations helped create an image of the NIP as an interesting, yet in no way controversial object of policy. More specifically, one of the speakers suggested that the case of vaccination opposition was a hopeless one to tackle, thereby engaging in the kind of positioning work discussed above: regarding vaccination, one is either *in favor* or *against* it, the latter being an irrational and marginal position. The recent recommendation by the National Bioethics Commission (*Bioethikkommission*) to consider compulsory vaccination for health workers against a variety of infectious diseases further speaks to the strengthening of the policy discourse on vaccination (Bioethikkommission, 2015).

In sum, it seems that the initial failure to problematize screening in favor of (its combination with) primary prevention was a blessing in disguise for policymakers: While initially, the HPV vaccine seemed a costly, uncertain technology to *save women's lives*, it ultimately helped launch a renewed discourse on vaccination. In the ongoing embedding of the vaccine, chronic weaknesses of the NIP, such as divergent vaccination registers, were concealed.

## 5. Conclusion

The HPV vaccine had to be *adapted*, in order to become *good enough* in Austria. At first, the vaccine brought about a *problematization* of the Pap smear, questioning the *rightness* of not only this tool, but the invested *jobs*, or *social worlds* (Clarke and Star, 2008) that risked being shifted from screening to primary prevention. Yet the vaccine failed to convince as a tool to “save women's lives” – thus the storyline of its proponents at the time in 2007. In the second phase of HPV policy, labeled *enrollment* here, officials and clinical experts tinkered with the vaccine and its policy context and coordinated new alliances. The vaccine became gender-neutral, and sexuality was backgrounded by having pediatricians administer the vaccine. The *disenrollment* of gynecologists also functioned to disassociate the vaccine from allegedly biased researchers and profit-seeking vaccine manufacturers. Third, a new storyline – that of “protecting children and saving lives” functioned to conceal previous and persistent dissent that continues to shape the uneasy *embedding* of the vaccine against the backdrop of chronic instabilities (Singleton, 1998) of both screening and prevention infrastructures. The uneasy embedding of the two competing tools – Pap and HPV vaccine – remains vulnerable to future problematization. For the translation (Callon, 1986) explored here is, of course, an ongoing process, and much like the Pap smear (Casper and Clarke, 1998: 276), the HPV vaccine is not particularly right or wrong, but just *good enough*.

This study speaks to STS and policy scholarship addressing the coproduction of tools (Clarke and Fujimara, 1997), technologies, and social orders (Jasanoff, 2004). Across countries, the introduction of the vaccine brought up new and old questions of *rightness*. Yet in our case, the HPV vaccine did not become a public controversy, but a vehicle to achieve overarching political aims to strengthen the NIP and to launch a policy discourse on vaccination that marginalizes opposition. This finding stands in interesting contrast to HPV policy in the USA (Epstein, 2010), where the inclusion of boys, in fact, *re*-sexualized the vaccine as it revealed opportunities to protect gay men against anal cancer. The absence of this debate in Austria may in part be explained by the exclusion of men in the feminist and health economic discourses presented here, and by the absence of gay health advocacy in the present case. Finally, Austrian policymakers, likely having observed the earlier HPV controversies in North America, had little interest in publicizing the importance of protecting against anal cancer, given its “indiscussable” nature (Epstein, 2010).

This case study reveals the need for in-depth research of how new medical technologies are made “good enough” across different political contexts, how these transformative processes vary, and with what social and material effects. To health policy analysts, our findings regarding the *solidity* of political (infra)structures as well as the marginalization of civic dissent are particularly relevant and warrant further comparative analyses (Nathanson, 1996). Finally, policymakers concerned with low vaccine uptake in Austria may wish to invest in data collection on vaccine behavior to get a better understanding of how vaccination policy is tinkered with and translated across different social worlds, including medical professionals and private homes.
